# Protective effect of activated charcoal against progression of chronic kidney disease: A randomized clinical study

**DOI:** 10.25122/jml-2023-0128

**Published:** 2023-09

**Authors:** Waleed Khaild Rahman, Ihsan Salah Rabea, Mudhfar Mohammed Meizel

**Affiliations:** 1 Al-Diwaniyah Health Office, Ministry of Health and Environment, Al-Diwaniyah, Iraq; 2 Department of Clinical Pharmacy, Faculty of Pharmacy, University of Kufa, Al-Najaf, Iraq; 3 Department of Medicine, Medical College, Alqadisiyah University, Al-Diwaniyah, Iraq

**Keywords:** activated charcoal, CKD, urea, phosphorus, uremic toxin

## Abstract

Chronic kidney disease (CKD) is a non-reversible and progressive disease affecting the kidneys, significantly impacting global public health. One of the complications of chronic kidney disease is impaired intestinal barrier function, which may allow harmful products such as urea to enter the bloodstream and cause systemic inflammation. This study aimed to investigate whether supplementation with activated charcoal could reduce uremic toxins in patients with end-stage renal disease (ESRD). The study was a randomized clinical trial conducted at the Dialysis Center of al Diwaniyah Medical Hospital in the Diwaniyah Governorate. Eighty-two patients with ESRD on regular hemodialysis were enrolled, with 15 patients receiving oral supplementation with activated charcoal in addition to standard care and 13 patients receiving only standard care. Blood samples were collected at baseline and after eight weeks, and several biomarkers were measured, including estimated glomerular filtration rate (eGFR), creatinine, urea, phosphorus, albumin, and indoxyl sulfate. The results showed a significant reduction in both serum urea and serum phosphorus levels after eight weeks of oral-activated charcoal treatment. However, the other biomarkers were not affected by the treatment. In conclusion, the use of oral-activated charcoal for eight weeks in Iraqi patients undergoing maintenance hemodialysis improved urea and phosphorus levels.

## INTRODUCTION

Chronic kidney disease (CKD) is a progressive and irreversible syndrome that results in an inability to maintain metabolic, fluid, and electrolyte balance, thereby causing uremia or azotemia [1, 2]. CKD is marked by structural damage to nephrons that cannot be reversed [3] and is characterized by either abnormal albumin excretion or decreased kidney function, as measured by the glomerular filtration rate (GFR) over three months or more [4, 5]. If left untreated, CKD can progress to end-stage renal disease (ESRD) and necessitate renal replacement therapy (RRT). CKD is a global public health concern with significant morbidity, mortality, and economic burden [6]. The estimated global prevalence of CKD is 8-16%, with the highest rates observed in the United Kingdom (UK) and Singapore (both 16%), while in the United States, CKD affects 15% of the adult population, with approximately 1.9 million individuals receiving renal replacement therapy [7].

In CKD, there is a gradual loss of functional nephrons, which can be caused by primary kidney disease or secondary complications arising from systemic disorders, such as hypertension or diabetes mellitus. In addition, acute kidney injury can cause irreversible damage. In CKD, the colon adapts, assuming the role of the primary excretory organ responsible for maintaining bodily homeostasis. However, this adaptation can have severe effects on the gut environment. In individuals with CKD, serum urea accumulates, leading to an increased presence of urea within the intestinal lumen. Urease-producing bacteria then hydrolyze urea into ammonia and ammonium hydroxide, which increases intestinal pH, mucosal irritation, and structural changes to the gut barrier [8-10]. These changes contribute to the phenomenon known as “leaky gut” which facilitates the potential translocation of bacteria and toxins from the gut into the systemic circulation. This, in turn, can drive chronic inflammation, unfavorable cardiovascular consequences, and the progressive advancement of CKD [11-14].

Activated charcoal operates as an adsorbent within the gastrointestinal (GI) tract, effectively trapping various chemicals. These captured substances are then retained within the charcoal matrix, preventing or reducing their absorption into the bloodstream [15-17]. Orally administered activated charcoal remains unchanged in its form within the gastrointestinal tract as it does not undergo absorption through the intestinal lumen.

Activated charcoal and protein-limited diets are utilized to manage uremic symptoms in patients with diverse stages of renal disease. Through binding with urea and other urine toxins, activated charcoal effectively eliminates them via the feces [18]. Furthermore, dialysis efficiency can be improved by sorbents that remove waste products, such as indoxyl sulfate, urea, and other urinary toxins, as indicated by research [19].

Studies have been carried out to reduce the flow of uremic toxins from the gut to slow the progression of CKD [20]. Activated charcoal has been found to effectively eliminate waste products such as urea, indoxyl sulfate, and other urinary toxins, thereby improving dialysis [21]. According to research on animal models of chronic renal disease, activated charcoal can decrease oxidative stress, inflammation, and the pace of renal disease progression [22]. This is primarily due to its ability to reduce the formation and absorption of indoxyl sulfate and p-cresol sulfate [23]. The present study aimed to investigate the potential of activated charcoal supplementation in reducing uremic toxins in patients with end-stage renal disease (ESRD).

## Material and Methods

A randomized clinical study was conducted at the Dialysis Center of al Diwaniyah Medical Hospital in the Diwaniyah Government in Iraq from Oct 1^st^, 2022, to Jan 20^th^, 2023. The study enrolled 42 patients with ESRD who were undergoing regular hemodialysis. Of these, 21 patients were assigned to receive oral supplementation with activated charcoal from AMS company in addition to standard care, while the other 21 patients received only standard care. Blood samples were collected at baseline and after eight weeks to measure parameters such as estimated glomerular filtration rate (eGFR), creatinine, urea, phosphorus, albumin, and indoxyl sulfate. Data were recorded using a data extraction sheet.

The primary objective of the study was to investigate the association between renal function tests and the use of activated charcoal. As a secondary objective, the present study explored whether demographic characteristics impacted the protective effect of activated charcoal. The inclusion criteria for the study were patients aged >18 and <75 years old, undergoing regular hemodialysis for at least one month, of both genders, able to communicate in Arabic language or through their caregiver, and able to provide informed consent. Patients who did not meet these criteria were excluded from the study.

### Statistical analysis

The statistical software SPSS version 23 and Microsoft Office Excel 2010 were utilized to perform statistical analysis. Categorical variables were represented as percentages and counts. To determine if quantitative variables exhibited normal distribution, the Kolmogorov-Smirnov test was used. Numeric variables with a normal distribution were expressed as mean and standard deviation, while those without were expressed as median and inter-quartile range. These measures indicate central tendency and dispersion, respectively. The study employed thorough statistical analysis using three tests: (1) Chi-square test to determine the association between categorical variables, but only when expected counts were greater than 5 in 20% of cells or more, (2) Independent sample t-test to compare means between two groups if numeric variables were normally distributed, or Mann-Whitney U test if non-normally distributed, and (3) Paired t-test to compare means before and after treatment. The data was meticulously analyzed using these tests, displaying a rigorous approach to the study.

## RESULTS

**Figure 1:**
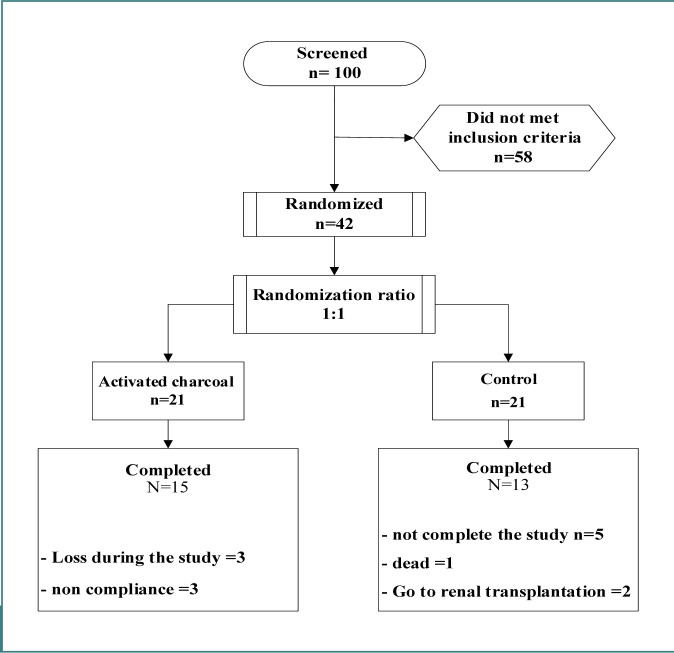
Patients distribution in the study, N = the number of patients

In the study, 100 dialysis patients were initially screened for eligibility, out of which 42 patients met the inclusion criteria, and 28 were eventually enrolled. The remaining 14 patients had different reasons for not completing the study. Figure 1 displays the distribution of these patients.

Table 1 presents the general characteristics of the patients enrolled in the study. The mean age of the control and activated charcoal groups were 46.08±12.79 years and 38.60±14.74 years, respectively (p=0.079), and there was no significant difference in the duration of dialysis since onset between the two groups. Similarly, the mean length of dialysis sessions and frequency of sessions per week showed no significant difference between the control and activated charcoal groups. There was also no significant difference in the gender distribution between the two groups. However, there was a significant difference in the residency distribution among the control and activated charcoal groups (p=0.037). Table 1 also displays the frequency distribution of patients according to body mass index and education level.

**Table 1:** General characteristics of patients enrolled in the present study

Characteristic	Control group n=13	Activated charcoal group n=15	p
**Age (years)**			
Mean ± SD	46.08±12.79	38.60±14.74	0.079 I NS
Range	25-63	18-67	
**Duration since dialysis (years)**			
Median (IQR)	4 (4.5)	2 (5)	0.298 M NS
Range	1-10	1-9	
**Session length (hour)**			
Mean ± SD	3.12±0.36	3.23±0.42	0.402 I NS
Range	2.5-4	3-4	
**Sessions per week**			
Mean ± SD	2.77±0.73	2.67±0.49	0.671 I NS
Range	1-4	2-3	
**Gender**			
Male	8 (61.5 %)	9 (60.0 %)	0.934 C NS
Female	5 (38.5 %)	6 (40.0 %)	
**BMI (kg/m^2^)**			
Underweight	6 (46.2 %)	5 (33.3 %)	†
Normal weight	4 (30.8 %)	5 (33.3 %)	
Overweight	2 (15.4 %)	3 (20.0 %)	
Obese	1 (7.7 %)	2 (13.3 %)	
**Residency**			
Urban	2 (15.4 %)	8 (53.3 %)	0.037 C *
Rural	11 (84.6 %)	7 (46.7 %)	
**Education level**			
Illiterate	5 (38.5 %)	2 (13.3 %)	†
Primary	7 (53.8 %)	4 (26.7 %)	
Secondary	0 (0.0 %)	4 (26.7 %)	
Tertiary	1 (7.7 %)	5 (33.3 %)	

n: number of cases; SD: standard deviation; IQR: inter-quartile range; BMI: body mass index; I: Independent samples t-test; M: Mann Whitney U test; C: chi-square test; NS: not significant; *: significant at p≤0.05; †: more than 20% of cells have expected count of more than 5

Table 2 displays the frequency distribution of patients and control subjects based on smoking and chronic medical illnesses. The control group and activated charcoal group had 1 (7.7%) and 3 (20.0%) smokers, respectively. The control group had 8 (61.5%) cases of essential hypertension, whereas the activated charcoal group had 12 (80.0%) cases. Urinary tract infection was found in 3 (23.1%) and 6 (40.0%) cases in the control and activated charcoal groups, respectively. Diabetes mellitus was found in 4 (30.8%) and 1 (6.7%) cases in the control group and activated charcoal group, respectively. The control group had 3 (23.1%) cases of ischemic heart disease and 1 (7.7%) case of heart failure, while the activated charcoal group had none. Renal stone was found in 1 (7.7%) and 2 (13.3%) patients in the control group and activated charcoal group, respectively. The control group had 1 (7.7%) case of epilepsy, while the activated charcoal group had none.

**Table 2:** The frequency distribution of patients and control subjects according to smoking and chronic medical illnesses

Characteristic	Control group n=13	Activated charcoal group n=15	p
Smoking	1 (7.7 %)	3 (20.0 %)	†
Essential hypertension	8 (61.5 %)	12 (80.0 %)	†
Urinary tract infection	3 (23.1 %)	6 (40.0 %)	†
Diabetes mellitus	4 (30.8 %)	1 (6.7 %)	†
Ischemic heart disease	3 (23.1 %)	0 (0.0 %)	†
Heart failure	1 (7.7 %)	0 (0.0 %)	†
Renal stone	1 (7.7 %)	2 (13.3 %)	†
Epilepsy	1 (7.7 %)	0 (0.0 %)	†

n: number of cases; †: more than 20% of cells have an expected count of more than 5

Table 3 displays the biochemical parameters prior to the initiation of treatment. The baseline estimated glomerular filtration rate (GFR) showed no significant difference between the control and activated charcoal groups, 6.54±2.03 ml/minute/1.73 m^2^ and 5.27±1.98 ml/minute/1.73m^2^, respectively (p=0.106). Baseline blood urea was also not significantly different between the control and activated charcoal groups, 131.15±32.85 mg/dl and 144.54±38.67 mg/dl, respectively (p=0.337). However, a significant difference was observed in baseline serum creatinine between the two groups, control and activated charcoal groups, 8.98±1.55 mg/dl and 11.19±2.69 mg/dl, respectively (p=0.015).

**Table 3:** Biochemical parameters before starting treatment

Characteristic	Control group n=13	Activated charcoal group n=15	p
**Estimated GFR ml/minute/1.73m^2^**			
Mean ± SD	6.54±2.03	5.27±1.98	0.106 I NS
Range	4.00-11.00	3.00-11.00	
**Blood urea mg/dl**			
Mean ± SD	131.15±32.85	144.54±38.67	0.337 I NS
Range	77.00-183.00	90.00-228.00	
**Serum creatinine mg/dl**			
Mean ± SD	8.98±1.55	11.19±2.69	0.015 I *
Range	6.75-11.43	5.54-15.50	
**Serum albumin g/dl**			
Mean ± SD	3.46±0.39	3.65±0.36	0.193 I NS
Range	2.70-4.10	3.00-4.10	
**Serum phosphorus mg/dl**			
Mean ± SD	4.82±1.36	6.10±2.56	0.118 I NS
Range	2.40-7.90	2.30-10.10	
**Serum Indoxyl sulfate ng/ml**			
Mean ± SD	519.18±139.17	435.25±167.96	0.166 I NS
Range	184.50-760.37	185.70-712.62	

n: number of cases; SD: standard deviation; GFR: glomerular filtration rate; I: Independent samples t-test; NS: not significant; *: significant at p≤0.05

Furthermore, baseline serum albumin showed no significant difference between the control and activated charcoal groups, 3.46±0.39 g/dl and 3.65±0.36 g/dl, respectively (p=0.193). Additionally, no significant difference was observed in baseline serum phosphorous between the control and activated charcoal groups, 4.82±1.36 mg/dl and 6.10±2.56 mg/dl, respectively (p=0.118). Lastly, there was no significant difference in baseline serum indoxyl sulfate between the control and activated charcoal groups, 519.18±139.17 ng/ml and 435.25±167.96 ng/ml, respectively (p=0.166).

Table 4 shows the comparison of mean differences in serum levels of biochemical markers among groups. There was no significant difference in the mean difference of estimated glomerular filtration rate (eGFR) among the groups (p=0.788). However, there was a significant difference in the mean difference of blood urea among groups, with the activated charcoal group showing the highest reduction level (p<0.001). There was no significant difference in the mean difference of serum creatinine (p=0.352) or serum albumin (p=0.824) among the groups. There was a significant difference in the mean difference of serum phosphorus, with the activated charcoal group showing the highest reduction level (p=0.013). Finally, there was no significant difference in the mean difference of serum indoxyl sulfate among the groups (p=0.324).

**Table 4:** Comparison of mean difference of biochemical markers between the activated charcoal group and control group

Characteristic	Control group n=13	Activated charcoal group n=15	p
**Estimated GFR**			
Mean±SD	0.08±2.72	-0.13±1.19	0.788 I NS
Range	-6.00-4.00	-3.00-1.00	
**Blood Urea**			
Mean±SD	47.33±41.73	-15.43±33.71	<0.001 I***
Range	-63.92-99.00	-63.00-56.00	
**Serum Creatinine**			
Mean±SD	1.09±4.71	-0.12±1.40	0.352 I NS
Range	-2.39-14.53	-1.77-2.26	
**Serum Albumin**			
Mean±SD	0.49±0.73	0.55±0.62	0.824 I NS
Range	-1.11-2.02	-0.40-1.81	
**Serum Phosphorus**			
Mean±SD	1.30±2.37	-0.85±1.91	0.013 I*
Range	-3.61-5.90	-5.30-1.70	
**Serum Indoxyl sulfate**			
Mean±SD	-93.42±166.82	-30.93±161.52	0.324 I NS
Range	-436.05-195.78	-353.11-161.84	

n: number of cases; SD: standard deviation; I: Independent samples t-test; NS: not significant; *: significant at p≤0.05; ***: significant at p≤0.001

## DISCUSSION

This study investigated whether oral activated charcoal could adsorb uremic toxins and improve renal function tests in patients with end-stage renal disease undergoing regular hemodialysis. The process, known as “intestinal dialysis” occurs when urea and other waste products bind to charcoal and are excreted in the feces, creating a concentration gradient for continuous diffusion [24]. The present study compared eGFR, serum urea, creatinine, serum albumin, serum phosphorus, and indoxyl sulfate levels in the groups at baseline and after eight weeks of follow-up.

The activated charcoal group showed a significant decrease in serum urea and phosphorus throughout the follow-up period. A study by Al-Shazly *et al*. [25] showed similar results over a 12-week follow-up period, where the activated charcoal group had a significant decrease in serum urea and phosphorus compared to the control group. Another study by Gao *et al*. [26] supported these findings, demonstrating that the phosphorus levels in the oral-activated charcoal group started to decline in the third month and remained lower than those in the placebo group throughout the experiment.

It is well known that the kidneys cannot excrete waste products such as urea and creatinine in end-stage renal disease. Instead, their excretion relies mainly on hemodialysis procedures [27]. As urea has a lower molecular weight than creatinine, it needs less hemodialysis time to be dialyzed [28]. This could explain the non-significant decrease in creatinine levels in both groups.

Regarding albumin levels, most end-stage renal disease patients suffer from hypoalbuminemia for several reasons, such as a low protein diet, protein loss through urine, and dialysate, especially with high flux filters. However, the current study found a non-significant decrease in albumin levels. This could be due to the frequent monitoring of albumin levels in both groups and intravenous albumin treatment was administered to patients with hypoalbuminemia. Additionally, the majority of dialysis patients suffer from malnutrition associated with anorexia and poor protein intake.

Furthermore, the study revealed an increase in eGFR in the control group and a decrease in the activated charcoal group, but the changes did not reach significant levels in either group. These insignificant changes may be due to the need for longer durations of activated charcoal use to show beneficial effects or a larger sample size [29].

The uremic toxin indoxyl sulfate is difficult to remove through regular hemodialysis, and even hemodiafiltration has limited success in removing it [30]. Various methods have been explored to address this challenge, including reducing the indoxyl sulfate concentration in the dialysate, increasing the dialysate flow rate, and employing larger dialyzers. However, the clinical effectiveness of these approaches remains uncertain [31, 32]. Limiting protein intake can reduce indoxyl sulfate production, and a low-protein diet supplemented with keto analogues has been shown to decrease serum indoxyl sulfate levels. Administration of activated charcoal has also been reported to lower indoxyl sulfate plasma levels in CKD patients [33-36].

The progressive decline of renal function in CKD leads to the accumulation of numerous uremic toxins, including indoxyl sulfate. This representative toxin builds up in the blood and tissues of patients with impaired kidney function [37, 38]. The most effective way to eliminate indoxyl sulfate is by binding it to activated charcoal and expelling it through feces. Accumulating this compound in blood can lead to cardiovascular diseases and decreased renal function in patients with CKD [39]. A dose-dependent decrease in serum indoxyl sulfate can be achieved through the use of adsorbents, but it is essential for patients to adhere to medication guidelines to avoid indoxyl sulfate accumulation and slow disease progression. It is, therefore, crucial for patients to comply with the prescribed dosage and treatment [40-43].

The current study has several limitations. Firstly, the sample size was restricted as only a small number of patients met the inclusion criteria out of 100 patients in the center. Secondly, the study was open-label due to concerns about black discoloration of stool, which may have influenced the tolerability and adverse effects. Thirdly, the study was novel, with few comparable studies or references. Finally, the study was conducted in a single center, and the results cannot be generalized to all centers treating hemodialysis patients in Iraq.

## CONCLUSION

In conclusion, oral activated charcoal showed promise in reducing uremic toxins in Iraqi patients on maintenance hemodialysis. However, larger, longer-term studies with multiple centers and a greater number of patients are needed to confirm its nephroprotective effect. Further research is also needed to determine its potential benefits in pre-dialyzed patients.

## Data Availability

The corresponding author can provide access to the database upon reasonable request.
